# Supplementary material for: Genetic diversity, genetic population structure and epidemiology of multidrug resistance Neisseria gonorrhoeae from Kenya
Source: PLoS One. 2026 Jan 5;21(1):e0339395. doi: 10.1371/journal.pone.0339395 (PMC12768347; doi:10.1371/journal.pone.0339395)
Supplement: S1 File — (DOCX) [file pone.0339395.s001.docx]

| Sample | Complete (%) | Single-copy (%) | Duplicated (%) | Fragmented (%) | Missing (%) | Total BUSCO groups |
| --- | --- | --- | --- | --- | --- | --- |
| GCA_014844795 | 95.2 | 95.2 | 0 | 2.4 | 2.4 | 124 |
| GCA_014844815 | 96.8 | 96.8 | 0 | 0.8 | 2.4 | 124 |
| GCA_014844835 | 94.4 | 94.4 | 0 | 2.4 | 3.2 | 124 |
| GCA_014844855 | 95.2 | 95.2 | 0 | 1.6 | 3.2 | 124 |
| GCA_014844875 | 96.8 | 96.8 | 0 | 0.8 | 2.4 | 124 |
| GCA_014844895 | 95.2 | 95.2 | 0 | 2.4 | 2.4 | 124 |
| GCA_014844915 | 96.8 | 96.8 | 0 | 0.8 | 2.4 | 124 |
| GCA_014844935 | 92.7 | 92.7 | 0 | 3.2 | 4 | 124 |
| GCA_014844955 | 96 | 96 | 0 | 0.8 | 3.2 | 124 |
| GCA_014844975 | 96 | 96 | 0 | 0.8 | 3.2 | 124 |
| GCF_014844795 | 95.2 | 95.2 | 0 | 2.4 | 2.4 | 124 |
| GCF_014844815 | 96.8 | 96.8 | 0 | 0.8 | 2.4 | 124 |
| GCF_014844835 | 94.4 | 94.4 | 0 | 2.4 | 3.2 | 124 |
| GCF_014844855 | 95.2 | 95.2 | 0 | 1.6 | 3.2 | 124 |
| GCF_014844875 | 96.8 | 96.8 | 0 | 0.8 | 2.4 | 124 |
| GCF_014844895 | 95.2 | 95.2 | 0 | 2.4 | 2.4 | 124 |
| GCF_014844915 | 96.8 | 96.8 | 0 | 0.8 | 2.4 | 124 |
| GCF_014844935 | 92.7 | 92.7 | 0 | 3.2 | 4 | 124 |
| GCF_014844955 | 96 | 96 | 0 | 0.8 | 3.2 | 124 |
| GCF_014844975 | 96 | 96 | 0 | 0.8 | 3.2 | 124 |
| SRR7534671 | 96.8 | 96.8 | 0 | 0 | 3.2 | 124 |
| SRR7534672 | 96.8 | 96.8 | 0 | 0 | 3.2 | 124 |
| SRR7534673 | 97.6 | 97.6 | 0 | 0 | 2.4 | 124 |
| SRR7534674 | 96.8 | 96.8 | 0 | 0 | 3.2 | 124 |
| SRR7534675 | 96.8 | 96.8 | 0 | 0 | 3.2 | 124 |
| SRR7534676 | 96.8 | 96.8 | 0 | 0 | 3.2 | 124 |
| SRR7534677 | 96.8 | 96.8 | 0 | 0 | 3.2 | 124 |
| SRR7534679 | 97.6 | 97.6 | 0 | 0 | 2.4 | 124 |
| SRR7534680 | 96.8 | 96.8 | 0 | 0 | 3.2 | 124 |
| SRR7534681 | 96.8 | 96.8 | 0 | 0 | 3.2 | 124 |
| SRR7534682 | 97.6 | 97.6 | 0 | 0 | 2.4 | 124 |
| SRR7534683 | 96.8 | 96.8 | 0 | 0 | 3.2 | 124 |
| SRR7534685 | 92.7 | 92.7 | 0 | 1.6 | 5.6 | 124 |
| SRR7534688 | 90.3 | 89.5 | 0.8 | 6.5 | 3.2 | 124 |
| SRR7534690 | 96.8 | 96.8 | 0 | 0 | 3.2 | 124 |
| SRR7534691 | 96.8 | 96.8 | 0 | 0 | 3.2 | 124 |
| SRR7534692 | 96.8 | 96.8 | 0 | 0 | 3.2 | 124 |
| SRR7534693 | 96.8 | 96.8 | 0 | 0 | 3.2 | 124 |
| SRR7534694 | 96.8 | 96.8 | 0 | 0 | 3.2 | 124 |
| SRR7534695 | 96.8 | 96.8 | 0 | 0 | 3.2 | 124 |
| SRR7534696 | 96.8 | 96.8 | 0 | 0 | 3.2 | 124 |
| SRR7534697 | 96.8 | 96.8 | 0 | 0 | 3.2 | 124 |
| SRR7534698 | 96.8 | 96.8 | 0 | 0 | 3.2 | 124 |
| SRR7534699 | 96.8 | 96.8 | 0 | 0 | 3.2 | 124 |
| SRR7534700 | 97.6 | 97.6 | 0 | 0 | 2.4 | 124 |
| SRR7534701 | 96.8 | 96.8 | 0 | 0 | 3.2 | 124 |
| SRR7534702 | 96.8 | 96.8 | 0 | 0 | 3.2 | 124 |
| SRR7534703 | 97.6 | 97.6 | 0 | 0 | 2.4 | 124 |
| SRR7534704 | 92.7 | 92.7 | 0 | 2.4 | 4.8 | 124 |
| SRR7534705 | 96.8 | 96.8 | 0 | 0 | 3.2 | 124 |
| SRR7534706 | 96.8 | 96.8 | 0 | 0 | 3.2 | 124 |
| SRR7534707 | 96.8 | 96.8 | 0 | 0 | 3.2 | 124 |
| SRR7534708 | 96 | 96 | 0 | 0 | 4 | 124 |
| SRR7534709 | 93.5 | 93.5 | 0 | 2.4 | 4 | 124 |
| SRR7534710 | 96.8 | 96.8 | 0 | 0 | 3.2 | 124 |

Supplementary material 1 (S1)
