# Supplementary material for: Genetic diversity, genetic population structure and epidemiology of multidrug resistance Neisseria gonorrhoeae from Kenya
Source: PLoS One. 2026 Jan 5;21(1):e0339395. doi: 10.1371/journal.pone.0339395 (PMC12768347; doi:10.1371/journal.pone.0339395)
Supplement: Attachment — Submitted filename: PONE-D-25-52951due 23rd.pdf [file pone.0339395.s002.pdf]

PLOS ONE

Genetic diversity, genetic population structure and epidemiology of multidrug resistance Neisseria gonorrhoeae from Kenya.

--Manuscript Draft--

**Manuscript Number:**	PONE-D-25-52951
**Article Type:**	Research Article
**Full Title:**	Genetic diversity, genetic population structure and epidemiology of multidrug resistance Neisseria gonorrhoeae from Kenya.
**Short Title:**	genetics driving epidemiology of multdrug resistant Neisseria gonorrhoeae
**Corresponding Author:**	DENNIS WAMALABE MUKHEBI, MsC<br>Pwani University<br>kilifi, KENYA
**Keywords:**	Gonorrhea, Neisseria gonorrhoeae, genetic diversity, multidrug resistance, epidemiology
**Abstract:**	Neisseria gonorrhoeae is the bacterial agent responsible for gonorrhea, a common sexually transmitted infection. The emergence of Neisseria gonorrhoeae multidrug-resistant (MDR) strains a presents a critical public health threat, especially due to its contribution to antimicrobial resistance (AMR) and treatment failure. Currently, resistance profiling of N. gonorrhoeae relies on phenotypic methods such as minimum inhibitory concentration (MIC) testing and identification of known resistance mutations. These are with limited application of genome-wide approaches to understand resistance evolution. The lack of genomic epidemiology data among Low- and Middle-Income Countries (LMICs) regions such as Kenya, hampers effective AMR tracking and designation of evidence-based, targeted treatments. This study aims to investigate the genetic diversity, population structure, and recombination dynamics of MDR N. gonorrhoeae isolates from Kenya using whole-genome SNP analysis. A total of 92 genomes (72 FASTQ reads and 20 assembled genomes) were retrieved from NCBI. De novo assembly, identification of AMR genes and variant calling were conducted, followed by Principal Component Analysis (PCA), Neighbor-Net clustering, nucleotide diversity (π), and linkage disequilibrium (LD) decay analysis to assess population structure and recombination patterns. Our results revealed a predominant genetic cluster with several divergent outlier strains, indicating moderate population differentiation. Nucleotide diversity varied across the genome, with distinct recombination hotspots. LD decay occurred rapidly within the first 1000 bp, suggesting high recombination rates. These results indicate that recombination plays a pivotal role in shaping genetic variability and AMR evolution in N. gonorrhoeae populations. The absence of strong geographic structure further implies that transmission dynamics, rather than regional isolation, drive the spread of resistance. In conclusion, genome-wide SNP analysis offers valuable insights into the genetic diversity and populations structure of MDR N. gonorrhoeae in Kenya. These findings support the integration of genomic surveillance into national strategies for antimicrobial resistance control. This study reveals how genetic analysis can guide better strategies to track and control drug-resistant gonorrhea in Kenya.
**Order of Authors:**	DENNIS WAMALABE MUKHEBI, MsC
	HENRY KIEMA MUSEE, BSC
**Additional Information:**	
**Question**	**Response**
**Financial Disclosure**<br><br>Enter a financial disclosure statement that describes the sources of funding for the work included in this submission. Review the submission guidelines for detailed	The author(s) received no specific funding for this work.

Powered by Editorial Manager® and ProduXion Manager® from Aries Systems Corporation

requirements. View published research articles from *PLOS ONE* for specific examples.<br><br>This statement is required for submission and **will appear in the published article** if the submission is accepted. Please make sure it is accurate.<br><br>**Funded studies**<br>Enter a statement with the following details:<br>• Initials of the authors who received each award<br>• Grant numbers awarded to each author<br>• The full name of each funder<br>• URL of each funder website<br>• Did the sponsors or funders play any role in the study design, data collection and analysis, decision to publish, or preparation of the manuscript?<br><br>Did you receive funding for this work?	
**Competing Interests**<br><br>Use the instructions below to enter a competing interest statement for this submission. On behalf of all authors, disclose any competing interests that could be perceived to bias this work—acknowledging all financial support and any other relevant financial or non-financial competing interests.<br><br>This statement is required for submission and **will appear in the published article** if the submission is accepted. Please make sure it is accurate and that any funding sources listed in your Funding Information later in the submission form are also declared in your Financial Disclosure statement.<br><br>View published research articles from *PLOS ONE* for specific examples.	The authors declare no conflict of interest.

**NO authors have competing interests**<br><br>Enter: *The authors have declared that no competing interests exist.*<br><br>**Authors with competing interests**<br><br>Enter competing interest details beginning with this statement:<br><br>*I have read the journal's policy and the authors of this manuscript have the following competing interests: [insert competing interests here]*<br><br>* typeset	
**Ethics Statement**<br><br>Enter an ethics statement for this submission. This statement is required if the study involved:<br><br>• Human participants<br>• Human specimens or tissue<br>• Vertebrate animals or cephalopods<br>• Vertebrate embryos or tissues<br>• Field research<br><br>Write "N/A" if the submission does not require an ethics statement.<br><br>General guidance is provided below. Consult the submission guidelines for detailed instructions. **Make sure that all information entered here is included in the Methods section of the manuscript.**	N/A

**Format for specific study types**<br><br>**Human Subject Research (involving human participants and/or tissue)**<br>• Give the name of the institutional review board or ethics committee that approved the study<br>• Include the approval number and/or a statement indicating approval of this research<br>• Indicate the form of consent obtained (written/oral) or the reason that consent was not obtained (e.g. the data were analyzed anonymously)<br><br>**Animal Research (involving vertebrate animals, embryos or tissues)**<br>• Provide the name of the Institutional Animal Care and Use Committee (IACUC) or other relevant ethics board that reviewed the study protocol, and indicate whether they approved this research or granted a formal waiver of ethical approval<br>• Include an approval number if one was obtained<br>• If the study involved *non-human primates*, add *additional details* about animal welfare and steps taken to ameliorate suffering<br>• If anesthesia, euthanasia, or any kind of animal sacrifice is part of the study, include briefly which substances and/or methods were applied<br><br>**Field Research**<br><br>Include the following details if this study involves the collection of plant, animal, or other materials from a natural setting:<br>• Field permit number<br>• Name of the institution or relevant body that granted permission	
**Data Availability**<br><br>Authors are required to make all data underlying the findings described fully available, without restriction, and from the time of publication. PLOS allows rare exceptions to address legal and ethical concerns. See the PLOS Data Policy and FAQ for detailed information.	Yes - all data are fully available without restriction

Powered by Editorial Manager® and ProduXion Manager® from Aries Systems Corporation

A Data Availability Statement describing where the data can be found is required at submission. Your answers to this question constitute the Data Availability Statement and **will be published in the article**, if accepted.<br><br>**Important:** Stating 'data available on request from the author' is not sufficient. If your data are only available upon request, select 'No' for the first question and explain your exceptional situation in the text box.<br><br>Do the authors confirm that all data underlying the findings described in their manuscript are fully available without restriction?	
**Describe where the data may be found in full sentences. If you are copying our sample text, replace any instances of XXX with the appropriate details.**<br><br>• If the data are **held or will be held in a public repository**, include URLs, accession numbers or DOIs. If this information will only be available after acceptance, indicate this by ticking the box below. For example: *All XXX files are available from the XXX database (accession number(s) XXX, XXX.).*<br>• If the data are all contained **within the manuscript and/or Supporting Information files**, enter the following: *All relevant data are within the manuscript and its Supporting Information files.*<br>• If neither of these applies but you are able to provide **details of access elsewhere**, with or without limitations, please do so. For example:<br><br>*Data cannot be shared publicly because of [XXX]. Data are available from the XXX Institutional Data Access / Ethics Committee (contact via XXX) for researchers who meet the criteria for access to confidential data.*<br><br>*The data underlying the results presented in the study are available from (include the name of the third party*	The raw whole genome sequences of Neisseria gonorrhoeae used in this project were deposited in the NCBI GenBank under BioProject PRJNA481622 (for the data which had only reads 72) and PRJNA660404 (for 10 genomes from Nairobi, Kenya)

and contact information or URL).<br>• This text is appropriate if the data are owned by a third party and authors do not have permission to share the data.<br><br>* typeset	
Additional data availability information:	

Manuscript

Click here to access/download;Manuscript;Manuscript_28_9_25.docx

Genetic diversity, genetic population structure and epidemiology of multidrug resistance *Neisseria gonorrhoeae* from Kenya.

Henry Kiema Musee[1], Dennis Wamalabe Mukkhebi[1,22]

[1]Department of Biochemistry and Biotechnology, Pwani University, Kilifi, Kenya
[2]Pwani University Biosciences Research Centre (PUBReC), Pwani University, Kilifi, Kenya

*Corresponding author Email: mukhebidennis95@gmail.com , d.mukhevi@pu.ac.ke

Abstract

Neisseria gonorrhoeae is the bacterial agent responsible for gonorrhea, a common sexually transmitted infection. The emergence of *Neisseria gonorrhoeae* multidrug-resistant (MDR) strains a presents a critical public health threat, especially due to its contribution to antimicrobial resistance (AMR) and treatment failure. Currently, resistance profiling of *N. gonorrhoeae* relies on phenotypic methods such as minimum inhibitory concentration (MIC) testing and identification of known resistance mutations. These are with limited application of genome-wide approaches to understand resistance evolution. The lack of genomic epidemiology data among Low- and Middle-Income Countries (LMICs) regions such as Kenya, hampers effective AMR tracking and designation of evidence-based, targeted treatments. This study aims to investigate the genetic diversity, population structure, and recombination dynamics of MDR *N. gonorrhoeae* isolates from Kenya using whole-genome SNP analysis. A total of 92 genomes (72 FASTQ reads and 20

assembled genomes) were retrieved from NCBI. De novo assembly, identification of AMR genes and variant calling were conducted, followed by Principal Component Analysis (PCA), Neighbor-Net clustering, nucleotide diversity (π), and linkage disequilibrium (LD) decay analysis to assess population structure and recombination patterns. Our results revealed a predominant genetic cluster with several divergent outlier strains, indicating moderate population differentiation. Nucleotide diversity varied across the genome, with distinct recombination hotspots. LD decay occurred rapidly within the first 1000 bp, suggesting high recombination rates. These results indicate that recombination plays a pivotal role in shaping genetic variability and AMR evolution in *N. gonorrhoeae* populations. The absence of strong geographic structure further implies that transmission dynamics, rather than regional isolation, drive the spread of resistance. In conclusion, genome-wide SNP analysis offers valuable insights into the genetic diversity and populations structure of MDR *N. gonorrhoeae* in Kenya. These findings support the integration of genomic surveillance into national strategies for antimicrobial resistance control. This study reveals how genetic analysis can guide better strategies to track and control drug-resistant gonorrhea in Kenya.

Keywords: *Gonorrhea*, *Neisseria gonorrhoeae*, genetic diversity, multidrug resistance, epidemiology

Introduction

Neisseria gonorrhoeae, is a causative agent of gonorrhea, and sexually transmitted bacterium affecting both male and female (Unemo & Shafer, 2014). It colonizes the genitourinary tract, establishing infections in the urethra and endocervix, hence triggering an inflammation. If untreated, *Neisseria gonorrhoeae* can lead to serious health complications, including disseminated gonococcal infection (DGI), pelvic inflammatory disease (PID), epididymitis in men, and adverse

pregnancy outcomes(Quillin & Seifert, 2018). Over the past 70 years, *Neisseria gonorrhoeae* has progressively developed resistance to several antibiotic classes, including penicillin, aminoglycosides, macrolides, and tetracycline, which were once effective treatment options. The declining efficacy of these antibiotics is largely attributed to genetic mutations within *Neisseria gonorrhoeae* Notably, resistance to tetracycline has been reported, likely exacerbated by the discontinuation of these drugs as a primary treatment option. The introduction of ciprofloxacin and other fluoroquinolones was initially effective in controlling *Neisseria gonorrhoeae* infection (Belland et al., 1994). However, due to the emergence of bacterial resistance, fluoroquinolones, including ciprofloxacin, are no longer considered reliable treatment options (Unemo & Shafer, 2014).

Resistance to ciprofloxacin and other fluoroquinolones has necessitated the use of third-generation cephalosporins, such as cefixime and ceftriaxone, due to their high efficacy even at lower doses. These antibiotics were initially recommended as the first-line treatment for *Neisseria gonorrhoeae* infections. However, despite their effectiveness, the bacterium has developed resistance mechanisms that compromise their efficacy. The global dissemination of resistant *Neisseria gonorrhoeae* strains has led to a significant public health challenge, with resistance spreading rapidly across populations. In Kenya, resistance among most available antibiotics to *Neisseria gonorrhoeae* isolates has been linked to mutations in the gyrA and parC genes, which confer fluoroquinolone resistance (Kivata et al., 2019). A study by Juma et al.,(2021) uncovered that ciprofloxacin resistance is associated with multiple mutations in both gyrA and parC. Although these studies have documented the emergence of resistance strains using phenotypic surveillance and identification of key mutations, there remains a significant knowledge gap in the

understanding of genomic mechanisms that drive AMR, in Africa, low- and middle-income countries (LMICs) such as Kenya.

In this study we conducted a genomic analysis of 56 *Neisseria gonorrhoeae* isolates from Kenya, all of which demonstrated antimicrobial resistance to more than two antibiotics. The research aimed at elucidating genetic diversity, evolutionary features, and the potential impact of epidemiological factors using genome-wide SNP analysis. These factors include geographical distribution, transmission dynamics, antibiotic usage patterns, and host demographics, on the development of antimicrobial resistance.

The 92 high quality genomes (72 FASTQ reads and 20 assembled genomes) were retrieved from NCBI. De novo assembly was applied to the reads and then subjected to the SNP calling. Principal Component Analysis (PCA), Neighbor-Net analysis, nucleotide diversity (π) and Linkage disequilibrium (LD) decay plots were used to characterize the genetic relationships, diversity and recombination. These resulted into detecting clustering patterns, evolutionary divergence, and recombination hotspots relevant to resistance evolution.

Our findings revealed a dominant genetic cluster among Kenyan isolates, accompanied by several genetically divergent outliers, which indicates a moderate population differentiation. The LD decay and nucleotide diversity analyses provided strong evidence of frequent recombination events, suggesting that genetic exchange plays a pivotal role in shaping resistance traits. Moreover, the absence of strong geographic structuring highlights the importance of transmission dynamics over regional isolation in driving resistance spread.

This study advances the understanding of how the genetic plasticity and recombination influences resistance among *Neisseria gonorrhoeae* populations. Therefore, it emphasizes on the need for genomic surveillance in the development of targeted, evidence-based interventions. Our research also demonstrates the value of genome-wide SNP data in informing public health policy and highlights the urgency of integrating genomic tools into AMR monitoring strategies in LMICs such as Kenya.

Material and methods

Genomic data retrieval

A total of 72 FASTQ reads were obtained from the NCBI database under BioProject accession PRJNA481622, along with 20 complete genome sequences retrieved from BioProject PRJNA660404.

Processing and determination of variants

The quality of the raw sequencing reads was evaluated using FastQC version 0.11.9. The low-quality bases with a Phred score below 20 and adapter sequences were trimmed using Trimmomatic PE v0.39 (Bolger et al., 2014). The cleaned reads underwent de novo assembly using SPAdes v4.0.0 (Prjibelski et al., 2020), with default assembly parameters. The genome quality was evaluated using QUAST version 5.2 (Gurevich et al., 2013), and BUSCO tool (Simão et al., 2015), to determine genome completeness, (Supplementary 1), with the neisseriales_odb10 database. The number of predicted genes and their functions were determined to identify genes associated with antibiotic resistance (Table 1). The assembled genomes were aligned to a reference genome (GenBank assembly number GCA_013030075.1) using Burrows-Wheeler Aligner (BWA)

v0.7.18-r1243 (Li, 2013). The reference genome was first indexed using bwa index command and samtools faidx. Each genome was aligned to the reference, by creating a batch processing script which generated a Sequence Alignment Map (SAM) for each genome. The SAM files were converted to Binary Alignment Map (BAM) and sorted using SAMtools v1.15.1 (Li et al., 2009). All sorted genomes were merged and indexed using samtools. Bcftools v1.16 mpileup command was used to call for the variants using the reference genome and the merged sorted bam file, to produce Variants.vcf file, which contained the variants. Low quality variants (QUAL< 20) were filtered out using bcftools vcftools v0.1.16 (Danecek et al., 2011).

Population genetic structure analysis

The population genetic structure was investigated through Principal Component Analysis (PCA) and hierarchical clustering, utilizing SNP genotype data derived from VCF files. The genotype data were numerically encoded to represent distinct alleles, and missing genotype values were imputed using column means to reduce potential bias (Supplementary 2). PCA was performed to reduce the dimensionality of the genotype data, enabling the visualization of genetic variation among samples in a two-dimensional space defined by the first two principal components (PC1 vs. PC2). The FST was calculated using vcftools to confirm the genetic differentiation between the clusters, which was visualized in python. The genetic relationships among the various genomes isolates was determined using a SNP distance matrix generated in PLINK version 1.9, then a hierarchical clustering based on Unweighted Pair Group Method with Arithmetic Mean (UPGMA). A minimum-spanning neighbor network of MDR-NG isolates was constructed from SNP pairwise distances. Edges represent the shortest set of connections linking all isolates without cycles, preserving overall distance relationships. Nodes are colored by sampling location

Genetic diversity analysis

Genetic diversity was evaluated using VCFtools and PLINK, with subsequent visualizations conducted in Python. Nucleotide diversity (π) was computed on a per-site basis using VCFtools, producing an output file containing π values (Table 2). To analyze genomic variation, the mean nucleotide diversity was calculated within 1000 bp sliding windows across the genome. Linkage disequilibrium (LD) was assessed using PLINK. A BED file was generated from the genotype data, which served as the input for calculating pairwise LD (r^2 values) between SNPs. The resulting LD values were visualized using Python to investigate patterns of LD decay relative to genomic distances.

Results

Processing and determination of variants

Variant calling process across all 56 genomes yielded a total of 50710 Single Nucleotide Polymorphisms (SNPS) (figure 1).

Population genetic structure analysis

Our analysis of the population genetic structure revealed distinct clustering patterns (figure 2). The principal components (PC1 and PC2) shows proportion of the total genetic variation, with PC1 67.59% and PC2 4.44% of the variance (Figure 2). The genomes outside the primary cluster, includes GCA_014844855.1, GCA_014844815.1, GCA_014844795.1, GCA_014844975.1, GCA_014844895.1, GCA_014844935.1, GCA_014844955.1, GCA_014844875.1, and GCA_014844915.1. The genome of others isolates shows close genetic relationships within the

primary cluster. The FST statistics indicated that most genomic regions had low FST ranging from 0 to 0.1 while it also showed distinct peaks of high FST of up to 0.8–0.9 (Figure 3). The Neighbor-Net network analysis revealed the genetic structure among the genome of the isolates (Figure 4). The split network showed a dominant, well-connected cluster, aligning with the clustering patterns in the PCA plot (Figure 2).

Nucleotide diversity (π) and patterns of LD

The nucleotide diversity (π) plot showed variations in genetic diversity across all genomes, with π values ranging from 0.0 to ~ 0.6 (Figure 5). Multiple peaks were observed at distinct genomic positions, indicating regions of notable genetic variation. The LD decay plot (Figure 6) reveals the relationship between LD (measured as r^2) and genomic distance. LD values and genomic distance were inversely proportional. The fitted decay curve (represented by the red line) had a rapid decline in LD within the first 1000 base pairs (bp), then a gradual decrease which stabilized beyond 2000 bp.

Discussion

This study offers a detailed analysis of the genetic population structure and diversity of multidrug-resistant *Neisseria gonorrhoeae* isolates from Kenya using genome-wide SNP analysis. Our findings provide valuable insights informing targeted treatment strategies to combat *Neisseria gonorrhoeae* infections within the Kenyan population. Key observations included genetic differentiation, evidence of recombination, and critical insights into the mechanisms underlying antibiotic resistance evolution. These findings highlight the transmissibility and persistence of

Neisseria gonorrhoeae in human populations, hence need for tailored interventions to address the growing challenge of antimicrobial resistance.

The PCA revealed distinct clustering patterns, showing the presence of genetically differentiated subpopulations which the Kenyan N gonorrhoeae isolates. A substantial portion of the genetic variation was summarized along the first two principal components, suggesting distinct patterns in the data. A predominant cluster encompassing most isolates, alongside a few genetically divergent outliers. This points to limited overall diversity with potential signals of distinct evolutionary origins or introductions from external sources. Despite these clusters, no strong geographic structure was observed, suggesting that genetic variation is not entirely location-dependent but could be shaped by other selective pressures, such as antibiotic use and recombination events. The absence of clear geographic structuring is consistent with the high mobility of *Neisseria gonorrhoeae* and its ability to spread across populations due to human migration and sexual behaviors (Nacht et al., 2020).

To investigate further the genetic differentiation between the main genetic cluster in the PCA results and the outlier samples indicated in the PCA plot (figure 2), a genome-wide FST analysis was conducted using Vcftools. The results (figure 3) revealed that while the majority of the loci showed a low genetic differentiation, represented by the regions with FST 0–0.1, several genomic regions displayed an elevated FST of 0.4. The peaks of the FST analysis plot reach as high as 0.8, which indicates that there are regions which are under divergent selection or horizontal gene transfer events, and may be driving the observed structure in the PCA (figure 2).

The Neighbor-Net analysis further supported our findings by revealing a well-connected primary cluster with several reticulations in the network. These reticulations suggest recombination events

and shared genetic ancestry between some isolates. The presence of longer branch lengths in certain isolates supports the idea of genetic divergence that may be associated with acquired resistance mutations (Chander et al., 2021).

Extensive evidence of recombination in the Kenyan N gonorrhoeae isolates is a key finding of our study. Linkage disequilibrium decay analysis showed a sharp decline in the LD between 500–2000 bp, indicating frequent recombination events breaking down genetic associations over increasing distance (Kivikoski et al., 2023). High LD values (~0.8–1.0) at ~0 bp suggest tight linkage from low recombination, but LD decays rapidly by ~500–2000 bp due to increased recombination. Beyond 2000–3000 bp, LD stabilized at lower levels (~0.3–0.4), suggesting that longer genomic distances are more frequently broken by recombination events, resulting in a random association of alleles. The observed rapid LD decay pattern provides strong evidence that recombination is a dominant force shaping genetic variation in MDR *Neisseria gonorrhoeae* Our results are consistent with findings by Manoharan-Basil et al.(2021), who reported that *Neisseria gonorrhoeae* undergoes frequent horizontal gene transfer, facilitating the acquisition of beneficial mutations, particularly those associated with antibiotic resistance (Fiore et al., 2020).

Analysis of the nucleotide diversity (π) supports the role of recombination in shaping genetic variation, as regions with high nucleotide diversity overlapped with those exhibiting low LD. These findings suggest that recombination actively contributes to the genetic diversification of *Neisseria gonorrhoeae* populations in Kenya. Recombination hotspots in the genome are reflected as peaks of high nucleotide diversity in the scatter plot, suggesting a potential role in driving the adaptive evolution of antimicrobial resistance. This pattern is critical for the bacterium, as it enables rapid evolution in response to selective pressures, such as antibiotic use. The findings

underscore that recombination, in combination with high mutation rates, plays a central role in facilitating the adaptive potential of *Neisseria gonorrhoeae* Unlike many other bacteria that primarily rely on clonal expansion, *Neisseria gonorrhoeae* possesses a highly dynamic genome, allowing it to survive and spread even with ongoing treatment. There is a strong interplay between recombination and genetic variability, which results in the evolutionary adaptation of the organism (Arenas et al., 2018).

The high recombination rates and genetic diversity observed in *Neisseria gonorrhoeae* have significant implications for its transmissibility and evolution of antibiotic resistance (Katale et al., 2020). The genetic exchange and dissemination are pertinent in influencing genetic diversity and high recombination rates. The clusters that are observed in the population structure analysis indicate *Neisseria gonorrhoeae* can rapidly transfer resistance mutations within the population and across populations. The genetic flexibility of *Neisseria gonorrhoeae* promotes the spread of resistance to antibiotics such as tetracycline, penicillin, and cephalosporins, largely due to its high adaptability (De Korne-Elenbaas et al., 2022). Recombination events contribute to increased transmissibility and complicate infection control by enhancing the pathogen's fitness and survival across diverse populations. This adaptability facilitates easier transmission between hosts and supports the persistence of multidrug-resistant *Neisseria gonorrhoeae* strains, making eradication efforts increasingly challenging (Unemo & Shafer, 2014). *Neisseria gonorrhoeae* readily adapts to new hosts, and its genetic composition and diversity indicate that it is easily transmitted across diverse populations (Gibbs & Meyer, 1996). These findings underscore the necessity for more targeted antibiotic strategies that consider the genetic diversity and population structure of the bacterium, rather than approaching it as a homogeneous entity. Insights into the population structure reveal how adaptive mechanisms have contributed to the emergence of new *Neisseria*

gonorrhoeae strains with diverse genetic compositions associated with antimicrobial resistance. High genomic plasticity of *Neisseria gonorrhoeae* complicates current treatment regimens as new resistant strains can emerge unpredictably, which highlights the urgent need for adaptive treatment strategies, including combination therapies and genomic surveillance-based interventions, to effectively manage and control the spread of resistant gonococcal strains.

Conclusion

Our study advances the understanding of antibiotic resistance in *Neisseria gonorrhoeae* by characterizing its genetic diversity and population structure through SNP analysis, and by providing new insights into its recombination dynamics. The LD analysis revealed a rapid decay over short genomic distances, indicating high recombination activity among the isolates. This recombination contributes to elevated genetic diversity and facilitates the spread of antimicrobial resistance traits. Consistently, nucleotide diversity peaks aligned with regions of rapid LD decay, reinforcing the conclusion that recombination is a key driver of resistance evolution. The finding of the nucleotide diversity has shown that high recombination rates observed in the genomes of *Neisseria gonorrhoeae* are the key drivers of antimicrobial resistance, which increases its transmissibility. The analysis of the population structure of *Neisseria gonorrhoeae* from Kenyan isolates revealed different populations, although with outliers, indicating an ongoing development of resistance strains. Future research should broaden the biogeographic scope of the Kenyan *Neisseria gonorrhoeae* isolates and incorporate environmental analysis to assess whether the diversity is influenced by the ecological factors.

References

Arenas, M., Araujo, N. M., Branco, C., Castelhano, N., Castro-Nallar, E., & Pérez-Losada, M. (2018). Mutation and recombination in pathogen evolution: Relevance, methods and controversies. *Infection, Genetics and Evolution*, *63*, 295–306. https://doi.org/10.1016/j.meegid.2017.09.029

Belland, R. J., Morrison, S. G., Ison, C., & Huang, W. M. (1994). *Neisseria gonorrhoeae* acquires mutations in analogous regions of gyrA and parC in fluoroquinolone-resistant isolates. *Molecular Microbiology*, *14*(2), 371–380. https://doi.org/10.1111/j.1365-2958.1994.tb01297.x

Bolger, A. M., Lohse, M., & Usadel, B. (2014). Trimmomatic: A flexible trimmer for Illumina sequence data. *Bioinformatics*, *30*(15), 2114–2120. https://doi.org/10.1093/bioinformatics/btu170

Chander, S., Garcia-Oliveira, A. L., Gedil, M., Shah, T., Otusanya, G. O., Asiedu, R., & Chigeza, G. (2021). Genetic Diversity and Population Structure of Soybean Lines Adapted to Sub-Saharan Africa Using Single Nucleotide Polymorphism (SNP) Markers. *Agronomy*, *11*(3), 604. https://doi.org/10.3390/agronomy11030604

Danecek, P., Auton, A., Abecasis, G., Albers, C. A., Banks, E., DePristo, M. A., Handsaker, R. E., Lunter, G., Marth, G. T., Sherry, S. T., McVean, G., Durbin, R., & 1000 Genomes Project Analysis Group. (2011). The variant call format and VCFtools. *Bioinformatics*, *27*(15), 2156–2158. https://doi.org/10.1093/bioinformatics/btr330

De Korne-Elenbaas, J., Bruisten, S. M., Van Dam, A. P., Maiden, M. C. J., & Harrison, O. B. (2022). The Neisseria gonorrhoeae Accessory Genome and Its Association with the Core Genome and Antimicrobial Resistance. *Microbiology Spectrum*, *10*(3), e02654-21. https://doi.org/10.1128/spectrum.02654-21

Fiore, M. A., Raisman, J. C., Wong, N. H., Hudson, A. O., & Wadsworth, C. B. (2020). Exploration of the Neisseria Resistome Reveals Resistance Mechanisms in Commensals That May Be Acquired by N. gonorrhoeae through Horizontal Gene Transfer. *Antibiotics*, *9*(10), 656. https://doi.org/10.3390/antibiotics9100656

Gibbs, C. P., & Meyer, T. F. (1996). Genome plasticity in *Neisseria gonorrhoeae*. *FEMS Microbiology Letters*, *145*(2), 173–179. https://doi.org/10.1111/j.1574-6968.1996.tb08574.x

Gurevich, A., Saveliev, V., Vyahhi, N., & Tesler, G. (2013). QUAST: Quality assessment tool for genome assemblies. *Bioinformatics*, *29*(8), 1072–1075. https://doi.org/10.1093/bioinformatics/btt086

Juma, M., Sankaradoss, A., Ndombi, R., Mwaura, P., Damodar, T., Nazir, J., Pandit, A., Khurana, R., Masika, M., Chirchir, R., Gachie, J., Krishna, S., Sowdhamini, R., Anzala, O., & Meenakshi, I. S. (2021). Antimicrobial Resistance Profiling and Phylogenetic Analysis of Neisseria gonorrhoeae Clinical Isolates From Kenya in a Resource-Limited Setting. *Frontiers in Microbiology*, *12*, 647565. https://doi.org/10.3389/fmicb.2021.647565

Katale, B. Z., Misinzo, G., Mshana, S. E., Chiyangi, H., Campino, S., Clark, T. G., Good, L., Rweyemamu, M. M., & Matee, M. I. (2020). Genetic diversity and risk factors for the transmission of antimicrobial resistance across human, animals and environmental compartments in East Africa: A review. *Antimicrobial Resistance & Infection Control*, *9*(1), 127. https://doi.org/10.1186/s13756-020-00786-7

Kivata, M. W., Mbuchi, M., Eyase, F. L., Bulimo, W. D., Kyanya, C. K., Oundo, V., Muriithi, S. W., Andagalu, B., Mbinda, W. M., Soge, O. O., McClelland, R. S., Sang, W., &

Mancuso, J. D. (2019). gyrA and parC mutations in fluoroquinolone-resistant Neisseria gonorrhoeae isolates from Kenya. *BMC Microbiology*, *19*(1), 76. https://doi.org/10.1186/s12866-019-1439-1

Kivikoski, M., Rastas, P., Löytynoja, A., & Merilä, J. (2023). Predicting recombination frequency from map distance. *Heredity*, *130*(3), 114–121. https://doi.org/10.1038/s41437-022-00585-3

Li, H. (2013). *Aligning sequence reads, clone sequences and assembly contigs with BWA-MEM* (No. arXiv:1303.3997). arXiv. https://doi.org/10.48550/arXiv.1303.3997

Li, H., Handsaker, B., Wysoker, A., Fennell, T., Ruan, J., Homer, N., Marth, G., Abecasis, G., Durbin, R., & 1000 Genome Project Data Processing Subgroup. (2009). The Sequence Alignment/Map format and SAMtools. *Bioinformatics*, *25*(16), 2078–2079. https://doi.org/10.1093/bioinformatics/btp352

Manoharan-Basil, S. S., Laumen, J. G. E., Van Dijck, C., De Block, T., De Baetselier, I., & Kenyon, C. (2021). Evidence of Horizontal Gene Transfer of 50S Ribosomal Genes rplB, rplD, and rplY in Neisseria gonorrhoeae. *Frontiers in Microbiology*, *12*, 683901. https://doi.org/10.3389/fmicb.2021.683901

Nacht, C., Agingu, W., Otieno, F., Odhiambo, F., & Mehta, S. D. (2020). Antimicrobial resistance patterns in *Neisseria gonorrhoeae* among male clients of a sexually transmitted infections clinic in Kisumu, Kenya. *International Journal of STD & AIDS*, *31*(1), 46–52. https://doi.org/10.1177/0956462419881087

Prjibelski, A., Antipov, D., Meleshko, D., Lapidus, A., & Korobeynikov, A. (2020). Using SPAdes De Novo Assembler. *Current Protocols in Bioinformatics*, *70*(1), e102. https://doi.org/10.1002/cpbi.102

Quillin, S. J., & Seifert, H. S. (2018). Neisseria gonorrhoeae host adaptation and pathogenesis. *Nature Reviews Microbiology*, *16*(4), 226–240. https://doi.org/10.1038/nrmicro.2017.169

Simão, F. A., Waterhouse, R. M., Ioannidis, P., Kriventseva, E. V., & Zdobnov, E. M. (2015). BUSCO: Assessing genome assembly and annotation completeness with single-copy orthologs. *Bioinformatics*, *31*(19), 3210–3212. https://doi.org/10.1093/bioinformatics/btv351

Unemo, M., & Shafer, W. M. (2014). Antimicrobial Resistance in Neisseria gonorrhoeae in the 21st Century: Past, Evolution, and Future. *Clinical Microbiology Reviews*, *27*(3), 587–613. https://doi.org/10.1128/CMR.00010-14

Declarations

Ethics approval and consent to participate

Not applicable

Consent for publication

Not applicable

Competing interests

The authors declare no conflict of interest.

Funding information

Not applicable

Authors' contributions

HKM performed the data acquisition, analysis and wrote the manuscript, DWM supervised the work, with data analysis, design and coordination, review the manuscript, and conceptualized the idea. All authors have read and agreed to the published version of the manuscript.

Data availability

The raw whole genome sequences of *Neisseria gonorrhoeae* used in this project were deposited in the NCBI GenBank under BioProject PRJNA481622 (for the data which had only reads 72) and PRJNA660404 (for 10 genomes from Nairobi, Kenya)

Legends

Figure 1: SNP count per sample
Number of SNPs identified across each of the 56 *N. gonorrhoeae* isolates with color code.

Figure 2: Principal component analysis (PCA) of MDR-NG isolates from Kenya
Clustering pattern showing one dominant group and several divergent outliers. The samples are coded with their location.

Figure 3: FST analysis between PCA cluster and outliers
Scatter plot of FST between cluster and outliers of PCA.

Figure 4: Neighbor network analysis of MDR-NG isolates from Kenya
A minimum-spanning neighbor network of MDR-NG isolates, constructed from SNP pairwise distances. Edges represent the shortest set of connections linking all isolates without cycles, preserving overall distance relationships. Nodes are colored by sampling location.

Figure 5: Nucleotide diversity (π) across the genome
Nucleotide diversity across the genomes with peaks indicating regions of high genetic variability.

Figure 6: Linkage disequilibrium (LD) decay plot for MDR-NG isolates
Rapid LD decay within 1,000 bp reflects high recombination rates. Plateau beyond 2,000 bp indicates stabilized allele associations.

Figure

Click here to access/download;Figure;MS Legend (2).pptx

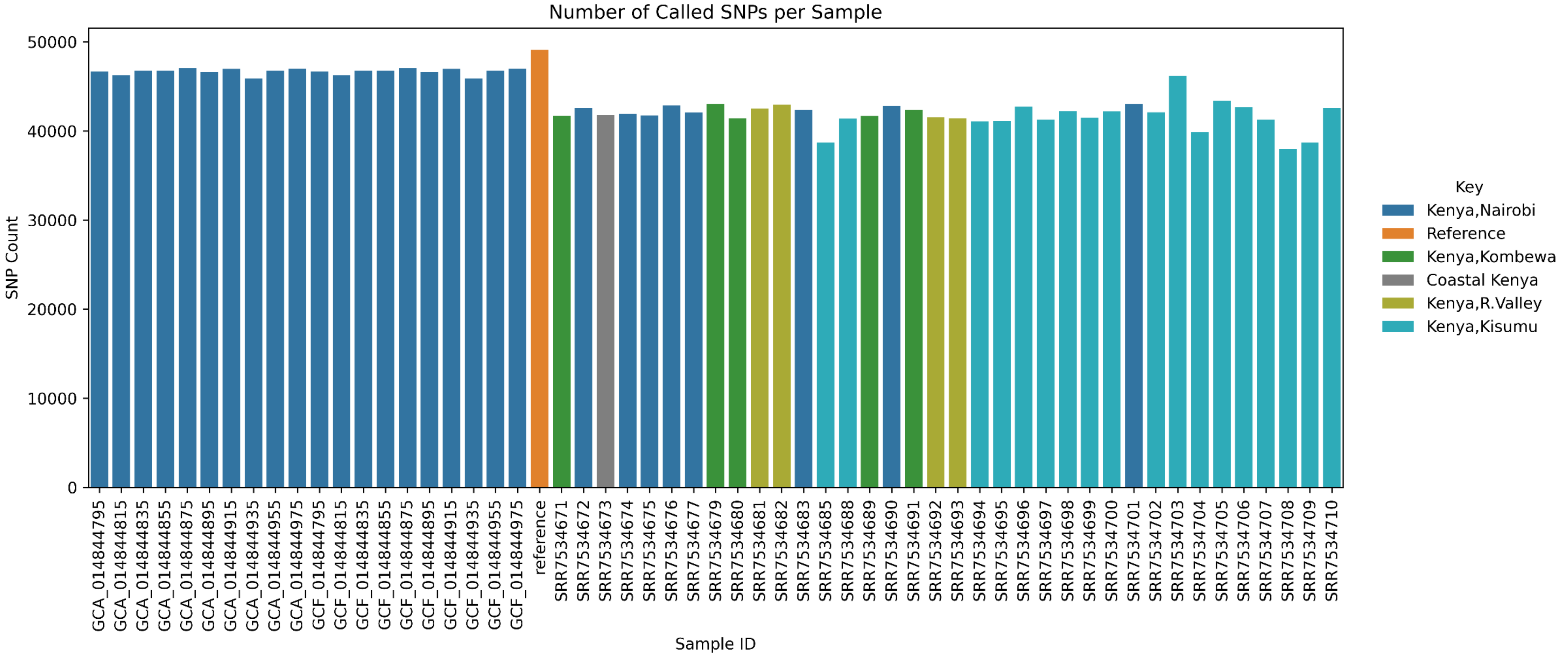

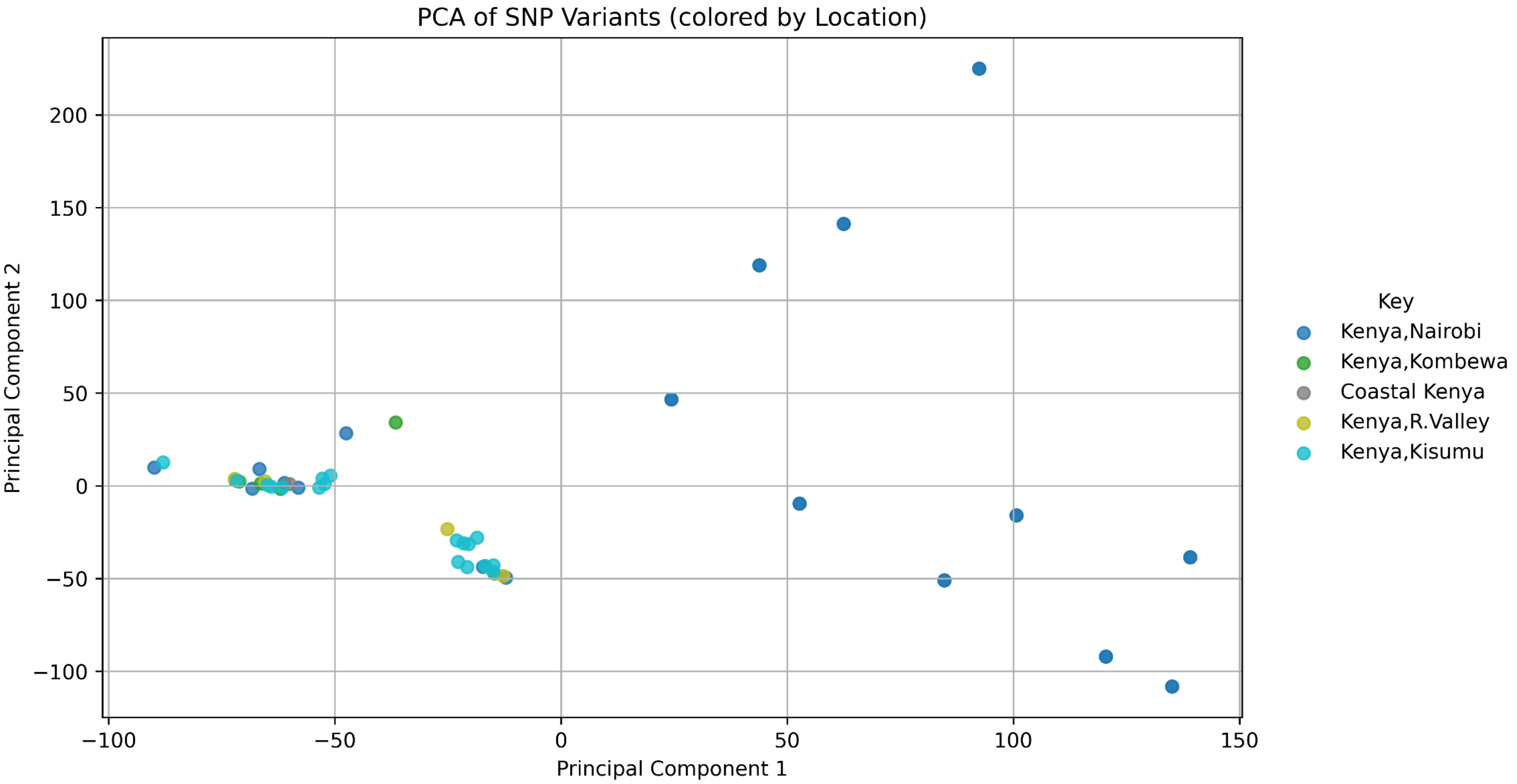

PCA of SNP Variants (colored by Location)
Principal Component 1
Principal Component 2
−100
−50
0
50
100
150
200
Key
Kenya,Nairobi
Kenya,Kombewa
Coastal Kenya
Kenya,R.Valley
Kenya,Kisumu

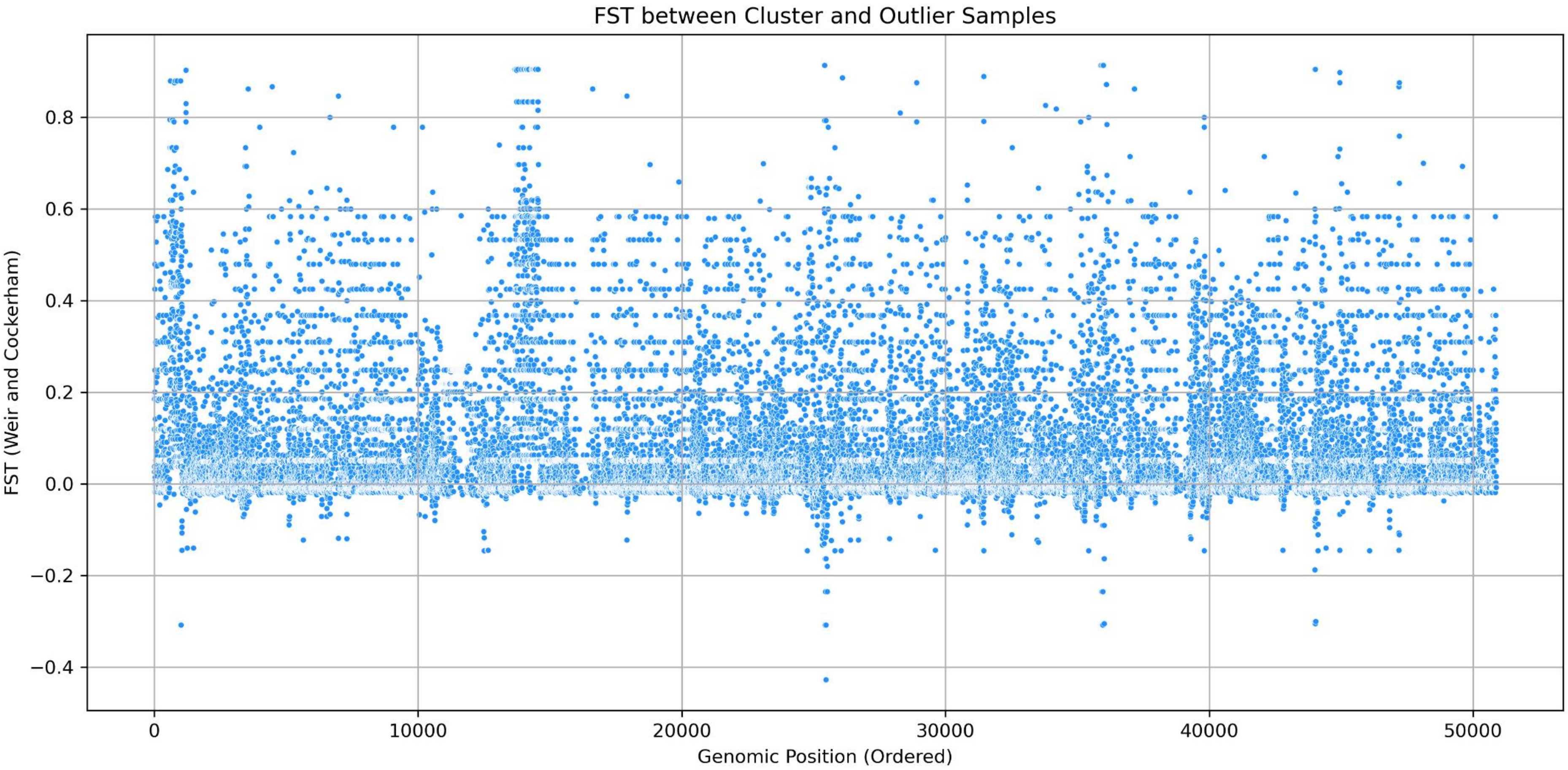

FST between Cluster and Outlier Samples
FST (Weir and Cockerham)
Genomic Position (Ordered)
0.8
0.6
0.4
0.2
0.0
−0.2
−0.4
0
10000
20000
30000
40000
50000

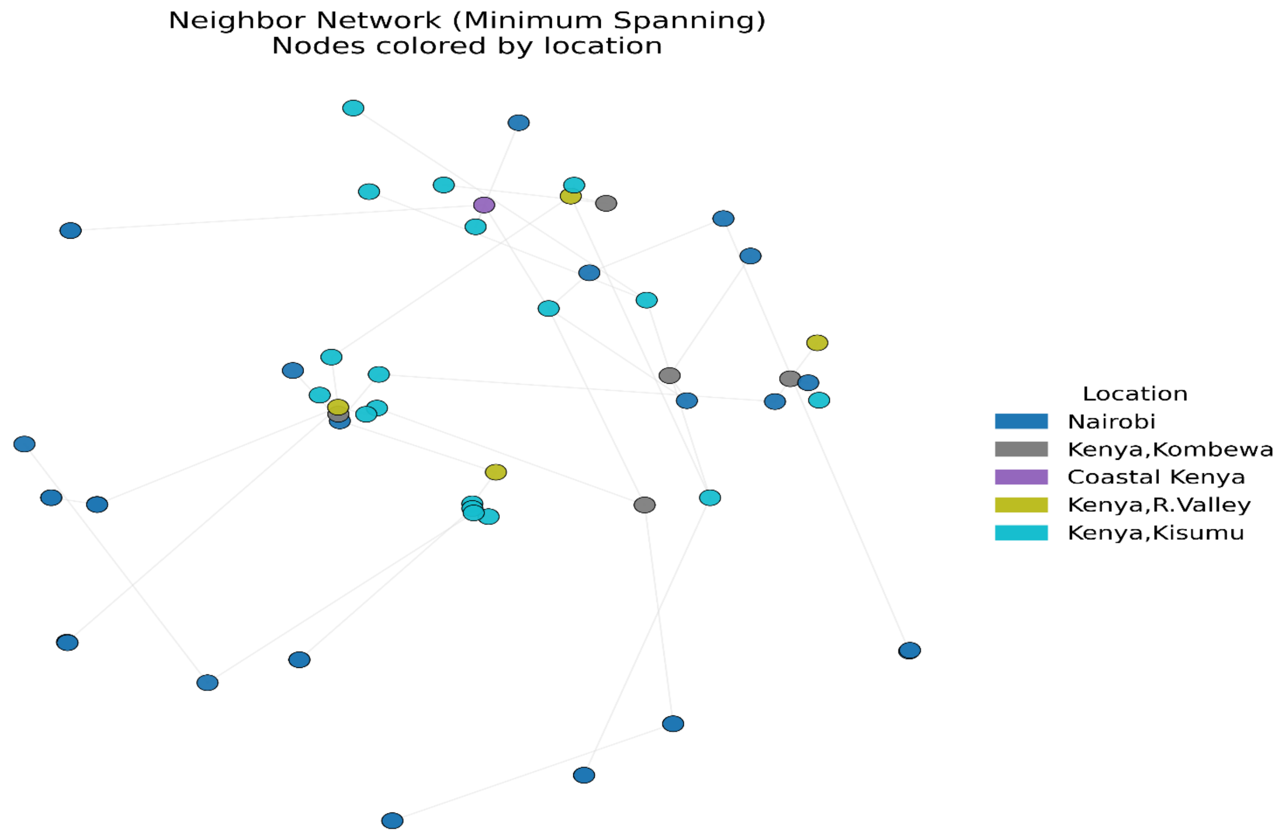
Neighbor Network (Minimum Spanning)
Nodes colored by location
Location
Nairobi
Kenya,Kombewa
Coastal Kenya
Kenya,R.Valley
Kenya,Kisumu

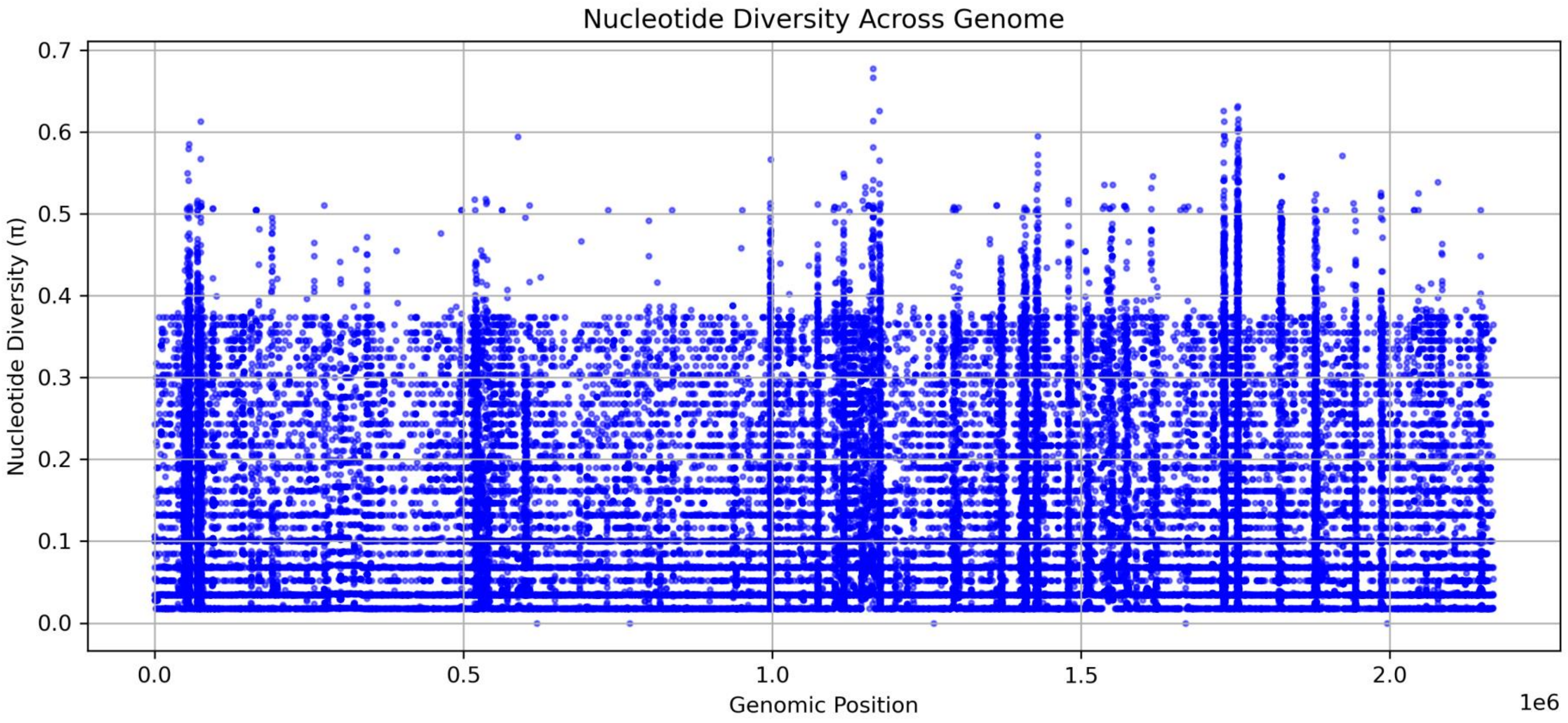
Nucleotide Diversity Across Genome
Nucleotide Diversity (π)
Genomic Position
1e6
0.0
0.1
0.2
0.3
0.4
0.5
0.6
0.7
0.0
0.5
1.0
1.5
2.0

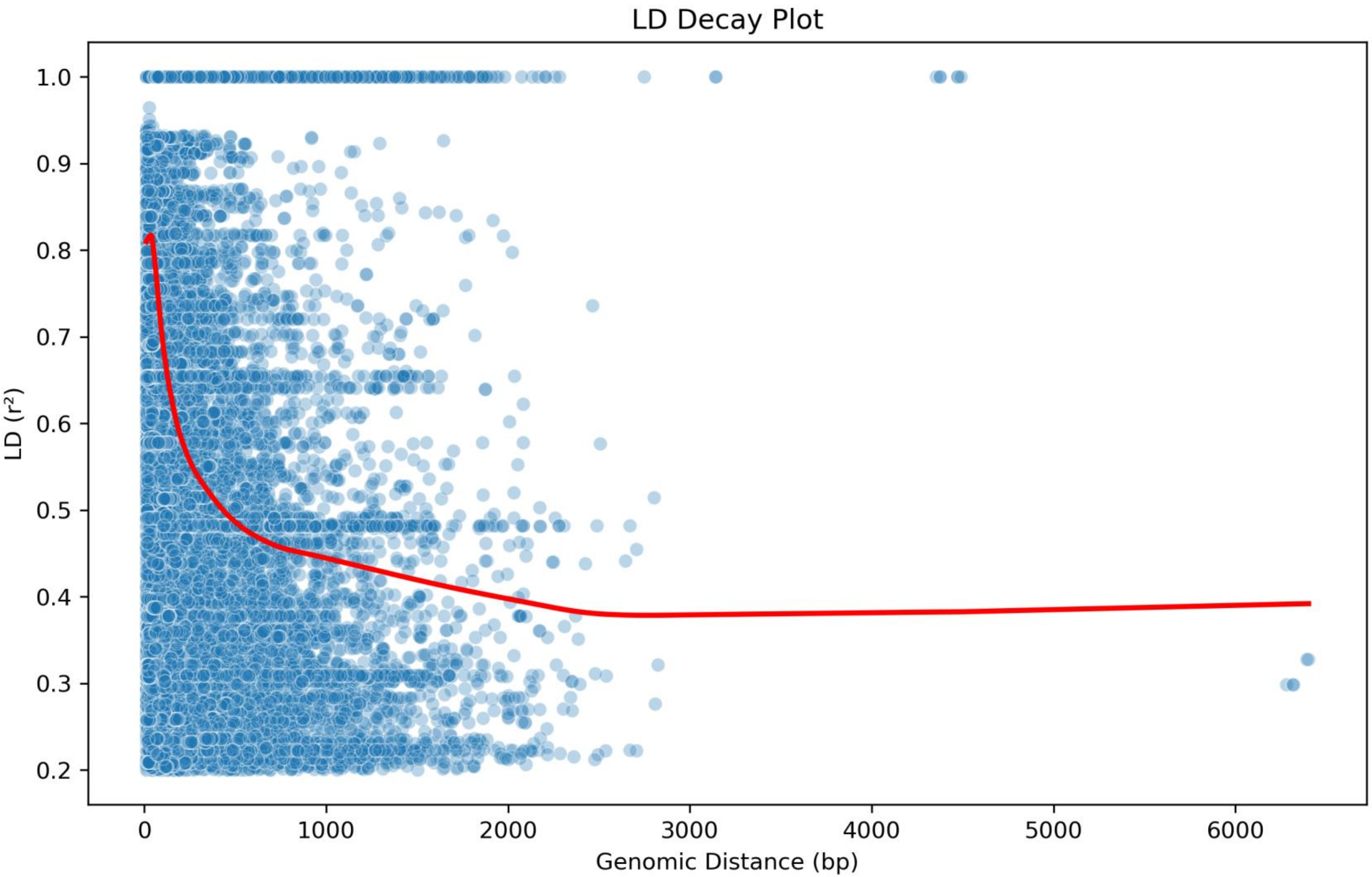
LD Decay Plot
LD (r²)
Genomic Distance (bp)
1.0
0.9
0.8
0.7
0.6
0.5
0.4
0.3
0.2
0
1000
2000
3000
4000
5000
6000

Click here to access/download
Supporting Information
Supplementary Material_1 Busco completness score.docx

Click here to access/download
Supporting Information
Supplementary 2 genotype_imputed_scaled.csv

Click here to access/download
Supporting Information
Table 1 number of genes (amr) per sample.docx

Click here to access/download
Supporting Information
Table 2 nucleotide diversity average values per sample.docx
