# Supplementary material for: Genetic diversity, genetic population structure and epidemiology of multidrug resistance Neisseria gonorrhoeae from Kenya
Source: PLoS One. 2026 Jan 5;21(1):e0339395. doi: 10.1371/journal.pone.0339395 (PMC12768347; doi:10.1371/journal.pone.0339395)
Supplement: Attachment — Submitted filename: Response to Reviewer_11_7_25.docx [file pone.0339395.s004.docx]

| **Reviewer Comment** | **Author Response** | **Changes Made in Manuscript (Manuscript_26_10_25_plos.docx)** |
| --- | --- | --- |
| **1. There is no geographical mapping of the isolates based on their genetic markers. The isolates have originated from various parts of Kenya. But genetic groupings area-wise have not been done. This will help delineate areas with increased AMR.** | **Addressed.** We performed rigorous statistical analysis to confirm significant regional differences in AMR gene burden, effectively delineating "hotspots." | **New Statistical Analysis & Figure:** **Kruskal–Wallis H test:** Confirmed significant variation in average AMR gene burden per isolate across regions (H = 10.87, *p* = 0.027).Dunn’s Post-hoc Test: Identified that isolates from Kisumu, Kombewa, and Rift Valley harbored significantly higher AMR gene counts compared to Nairobi (*p* < 0.05).New Boxplot: A new figure (referenced in the revised manuscript) displays AMR gene counts by region, annotated with significance bars, providing the requested geographical delineation. |
| **2. Specific genes coding for resistance have not been highlighted to show area-wise specific antibiotic resistance.** | **Addressed.** We expanded the analysis and discussion to specifically detail the regional distribution and type of key resistance mechanisms. | **Expanded Results & Discussion:** **Western Kenya** (Kisumu, Kombewa) showed a higher frequency and diversity of AMR variants, including those associated with β-lactam resistance (e.g., *penA* mutations) alongside core efflux and macrolide genes. Central/Coastal Kenya (Nairobi, Coastal Region) showed a tendency toward isolates carrying only the core efflux and macrolide determinants, suggesting different regional selection pressures on gene accumulation. |
| **3. No epidemiological correlation has been done.** | **Addressed.** We performed a critical correlation analysis between genomic diversity and AMR gene burden, which is a major new epidemiological finding. | **New Correlation and Abstract Update:** Correlation Finding: We established a clear positive correlation between high genetic diversity (π) and a significantly greater AMR gene burden. This finding is discussed as a key epidemiological mechanism: regions with higher genetic mixing are more likely to accumulate resistance genes. This finding is prominently included in the **Results and Discussion** sections. |
| **4. No population characteristics related to genomic diversity of the strains have been looked at.** | **Addressed.** We performed comprehensive population characteristics analysis using nucleotide diversity. | **New Population Genetics Analysis:** **Nucleotide Diversity (**π**)** was calculated for all isolates across regions. Regional Diversity: Isolates from Kisumu and Kombewa showed the highest π values (higher diversity/recombination), while Nairobi and Coastal isolates exhibited lower π values (more clonal populations). This analysis provides the essential population context for the epidemiological correlation detailed in point. |
| **5. Detail comments have been listed by track changes in the manuscript.** | **Addressed.** All track changes in the original manuscript and minor suggested edits have been reviewed and incorporated into the revised manuscript. | **Manuscript Revisions:** All identified comments and necessary text edits have been implemented in *Manuscript_26_10_25_plos.docx*. |
